# Prolonged silent carriage, genomic virulence potential and transmission between staff and patients characterize a neonatal intensive care unit (NICU) outbreak of methicillin-resistant *Staphylococcus aureus* (MRSA)

**DOI:** 10.1017/ice.2022.48

**Published:** 2022-03-21

**Authors:** Sharline Madera, Nicole McNeil, Paula Hayakawa Serpa, Jack Kamm, Christy Pak, Carolyn Caughell, Amy Nichols, David Dynerman, Lucy M. Li, Estella Sanchez-Guerrero, Maira S. Phelps, Angela M. Detweiler, Norma Neff, Helen Reyes, Steve A. Miller, Deborah S. Yokoe, Joseph L. DeRisi, Lynn Ramirez-Avila, Charles R. Langelier

**Affiliations:** 1 Division of Infectious Diseases, Department of Medicine, University of California, San Francisco, California; 2 Department of Hospital Epidemiology and Infection Prevention, University of California, San Francisco, California; 3 Genentech, Redwood City, California; 4 The Public Health Company, Santa Barbara, California; 5 Chan Zuckerberg Biohub, San Francisco, California; 6 Department of Laboratory Medicine, University of California San Francisco, California; 7 Department of Biochemistry and Biophysics, University of California, San Francisco, California; 8 Division of Pediatric Infectious Diseases and Global Health, Department of Pediatrics, University of California San Francisco, California

## Abstract

**Background:**

Methicillin-resistant *Staphylococcus aureus* (MRSA) is an important pathogen in neonatal intensive care units (NICU) that confers significant morbidity and mortality.

**Objective:**

Improving our understanding of MRSA transmission dynamics, especially among high-risk patients, is an infection prevention priority.

**Methods:**

We investigated a cluster of clinical MRSA cases in the NICU using a combination of epidemiologic review and whole-genome sequencing (WGS) of isolates from clinical and surveillance cultures obtained from patients and healthcare personnel (HCP).

**Results:**

Phylogenetic analysis identified 2 genetically distinct phylogenetic clades and revealed multiple silent-transmission events between HCP and infants. The predominant outbreak strain harbored multiple virulence factors. Epidemiologic investigation and genomic analysis identified a HCP colonized with the dominant MRSA outbreak strain who cared for most NICU patients who were infected or colonized with the same strain, including 1 NICU patient with severe infection 7 months before the described outbreak. These results guided implementation of infection prevention interventions that prevented further transmission events.

**Conclusions:**

Silent transmission of MRSA between HCP and NICU patients likely contributed to a NICU outbreak involving a virulent MRSA strain. WGS enabled data-driven decision making to inform implementation of infection control policies that mitigated the outbreak. Prospective WGS coupled with epidemiologic analysis can be used to detect transmission events and prompt early implementation of control strategies.

*Staphylococcus aureus* (*S. aureus*) remains a leading cause of hospital-acquired infections in the neonatal intensive care unit (NICU).^1^ Critically ill and preterm neonates are particularly vulnerable to invasive infections with methicillin-resistant *S. aureus* (MRSA), which confers significant morbidity, mortality,^2,3^ and financial costs.^3^ Nevertheless, the risk factors and mechanisms of transmission of MRSA in NICUs are incompletely understood.

Although MRSA colonization has been identified as a risk factor for the development of invasive infection,^4^ the specific factors contributing to transmission of MRSA between NICU patients are not well defined. Factors previously identified include environmental reservoirs because outbreak-associated MRSA strains can exhibit longer environmental persistence than sporadic-MRSA isolates.^5^ Parental colonization has been described as a source of MRSA both via vertical^6^ and horizontal^7,8^ transmission; however, these transmission events are usually self-limited and have not been implicated in outbreaks. Exposure to colonized healthcare personnel (HCP) or patients may play a role in precipitating outbreaks. In many hospitals, patients are tested for MRSA nares colonization upon admission; however, HCP generally are not.^9-11^

Here, we report an outbreak investigation of MRSA in a NICU in which HCP were identified as a potential key link in transmission and colonization. Whole-genome sequencing (WGS) further identified circulation of a highly virulent outbreak strain for at least 7 months prior to outbreak recognition in the NICU. We highlight an opportunity for prospective WGS in the surveillance setting to aid in the early identification of predominant circulating strains whose virulence may facilitate spread and invasive infection.

## Methods

### Setting

The University of California San Francisco (UCSF) NICU contains 58 beds and is located within the 183-bed UCSF Benioff Children’s Hospital.

### Patient consent

This study was approved by the UCSF Institutional Review Board (protocol 17-24056), which permitted WGS analysis of clinical isolates and review of clinical microbiology results as well as analysis of other electronic health record data.

### MRSA screening

Patients underwent weekly MRSA surveillance screenings through swabs of bilateral anterior nares, bilateral axillae, umbilical stump, and perirectal areas. HCP underwent a single screening evaluation that included a skin screening questionnaire and performed a self-swab of their bilateral anterior nares, bilateral axilla, and groin.

### Microbial culture

Bacterial cultures from patients with bacteremia were inoculated into BACTEC blood culture bottles (blood). Surveillance swabs and wound swabs were streaked onto Remel Spectra MRSA chromagar and incubated at 35°C for 24 hours (Thermo Scientific Spectra MRSA, Thermo Scientific, Waltham, MA). Plates showing colonies with characteristic color are reported as positive.

### Metagenomic sequencing

DNA was extracted from cultured isolates using the Zymo ZR Fungal/Bacterial DNA MiniPrep Kit (Zymo Research, Irvine, CA), 100 ng of DNA from each sample was then sheared with NEB fragmentase and used to construct sequencing libraries using the NEBNext Ultra II Library Prep Kit (New England Biolabs, Ipswich, MA). Adaptor ligated samples underwent 12 cycles of amplification with dual-indexing primers using the NEBNext Ultra II Q5 polymerase. Libraries were then quantified with Qubit (Thermo Scientific)and quality checked with a Bioanalyzer High Sensitivity DNA chip (Agilent, Santa Clara, CA). Samples were pooled and sequenced on an Illumina NextSeq instrument using a NextSeq 500/500 Mid Output kit v2.5 (300 cycles, Illumina, SanDiego, CO).

### Bioinformatic analyses

Raw sequences were analyzed using the SNP Pipeline for Infectious Disease (SPID) software.^12^ SPID aligned samples against *S. aureus* reference genome USA300 TCH1516 using minimap2, followed by samtools to perform an mpileup. Julia code was then run to call a consensus allele at each position, and the SNP instances were computed between every pair of samples. Phylogenetic analysis was performed using randomized axelerated maximum likelihood (RAxML).^13^ Phylogenetic trees were further visualized with ETE Python API software.^14^

ARGannot software was used to identify antimicrobial resistance genes from quality-filtered raw sequence data for each of the evaluated isolates.^15^ Genes with >99% gene coverage were included in the analyses. SCC*mec* elements were detected using SCC*mec*Finder.^16^ Multilocus sequence type (MLST) analysis was performed using MLST version 2.0 software.^17^ Detection of *S. aureus* virulence genes was performed using VirulenceFinder software.^18^

## Results

### Outbreak description

After 4 infants in the NICU with invasive MRSA infections were identified in an 8-day period, and an investigation was initiated. Retrospective review of microbiology data identified 2 additional positive MRSA cultures from infants in the same geographic zone of the NICU 2 months prior (Supplementary Table 1 online). NICU-wide surveillance screening of all infants and staff identified 16 other cases of MRSA colonization in asymptomatic infants or HCP (Supplementary Fig. 1 online). Review of prior MRSA isolates identified an isolate from an infant with multifocal MRSA infection 7 months prior (Supplementary Table 1 online, ID and 10B). In total, 23 MRSA isolates from 18 patients and 5 HCP were evaluated, and baseline characteristics of each study participant are listed in Supplementary Table 1 (online). Antimicrobial susceptibility testing (AST) demonstrated similarities across some isolates, suggestive of an outbreak (Supplementary Fig. 1 online).

### Genomic analyses

**Fig. 1.**
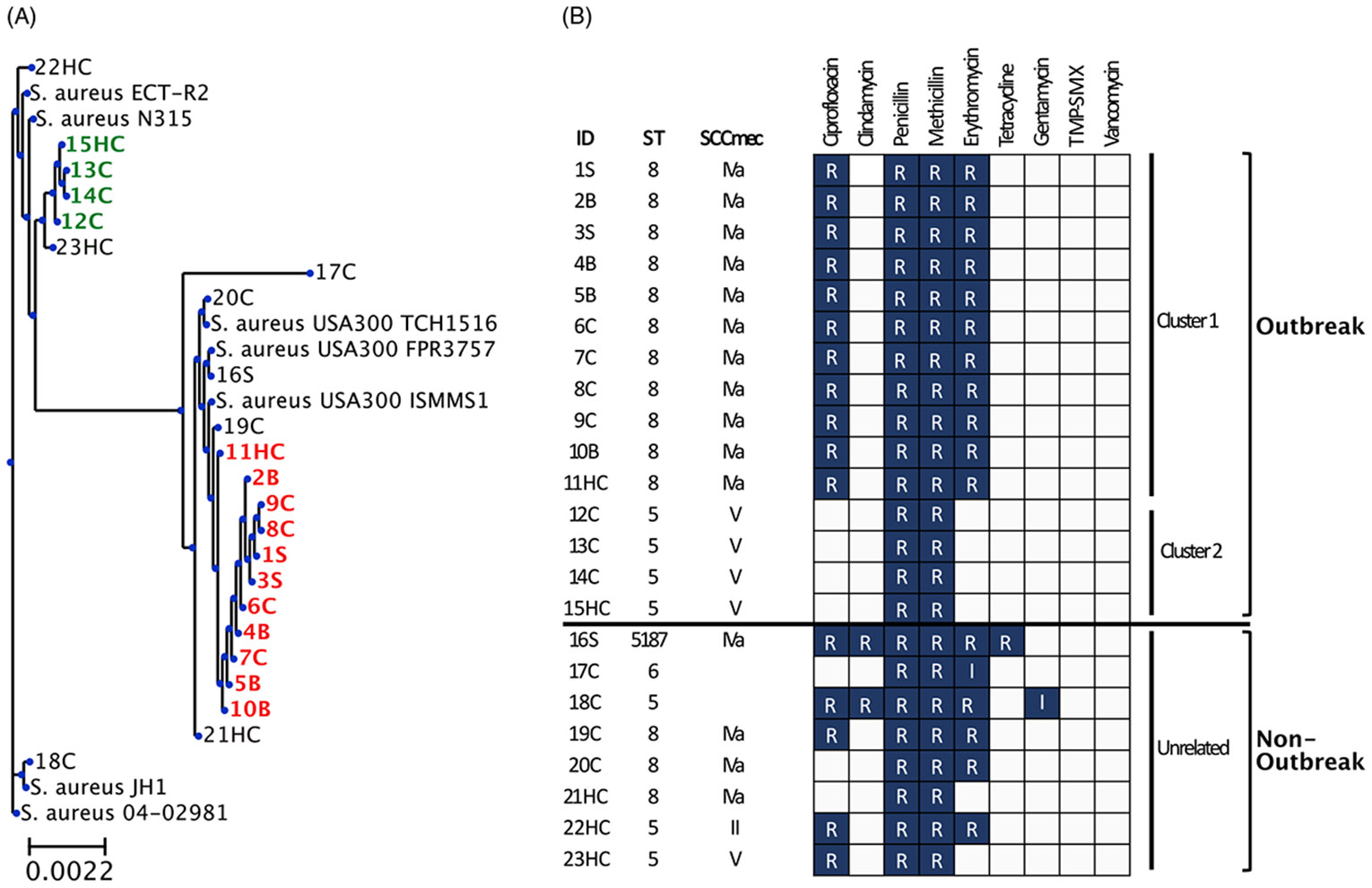
Phylogenetic analysis and phenotypic antibiogram of all sequenced MRSA cases. Isolates are identified by case number followed by H for healthcare personnel, B for bacteremia, S for skin and soft-tissue infection, and C for colonization. (A) Cases associated with clade 1 outbreak strain labeled red, and clade 2 outbreak strain labeled green. Reference *S. aureus* strains ECT-R2, N315, JH1, 04-02981, and USA 300 strains TCH1516, FPR3757, and ISMMS1, are shown. (B) Antimicrobial susceptibility pattern for all cases sequenced. Susceptibilities to antibiotics are denoted as resistant (R) or intermediate (I). Sequence type (MLST) and staphylococcal cassette chromosome (SCC) *mec* type are shown.

All isolates underwent WGS. Assembly by alignment against the chromosomal sequence of the National Center for Biotechnology Information (NCBI) reference strain *S. aureus* USA300 TCH1516 confirmed an outbreak composed of 2 genetically distinct clades (Fig. 1A). Pairwise single-nucleotide polymorphism (SNP) was used as a measure of genetic relatedness between isolates. A cutoff of 23 SNPs was used to define an isolate belonging to the same *S. aureus* phylogenetic clade.^19^ Phylogenetic analysis revealed and outbreak of 15 cases comprising 2 distinct phylogenetic clades; clade 1 was composed of 10 infants and 1 HCP (11), and clade 2 was composed of 3 infants and 1 HCP (Fig. 1A). Of the 23 cases, 8 were deemed unrelated to either of the 2 clades based on SNP distance.

Clade 1 exhibited marked genomic similarity between the 11 isolates that make up the clade with a median SNP difference of 3 (range, 0–6). Similarly, 4 isolates comprising clade 2 shared striking genetic similarity with a mean SNP difference of 7 (range 0–23). An isolate belonging to case ID 23EC was ~800 SNPs away from clade 2, thus was not likely part of a singular transmission event. Interestingly, clade 1 samples were more closely related to ST8 MRSA reference strains USA300 TCH 1516, ISMMS1, and FPR3757, with ~140 SNP differences. Clade 2 was more closely related to ST5 MRSA reference strain N315 with an ~700-SNP difference. MRSA reference strain N315 harbored a 17,000-SNP difference from clade 1, and ST8 MRSA reference strains carried an ~20,000-SNP difference from clade 2.

**Fig. 2.**
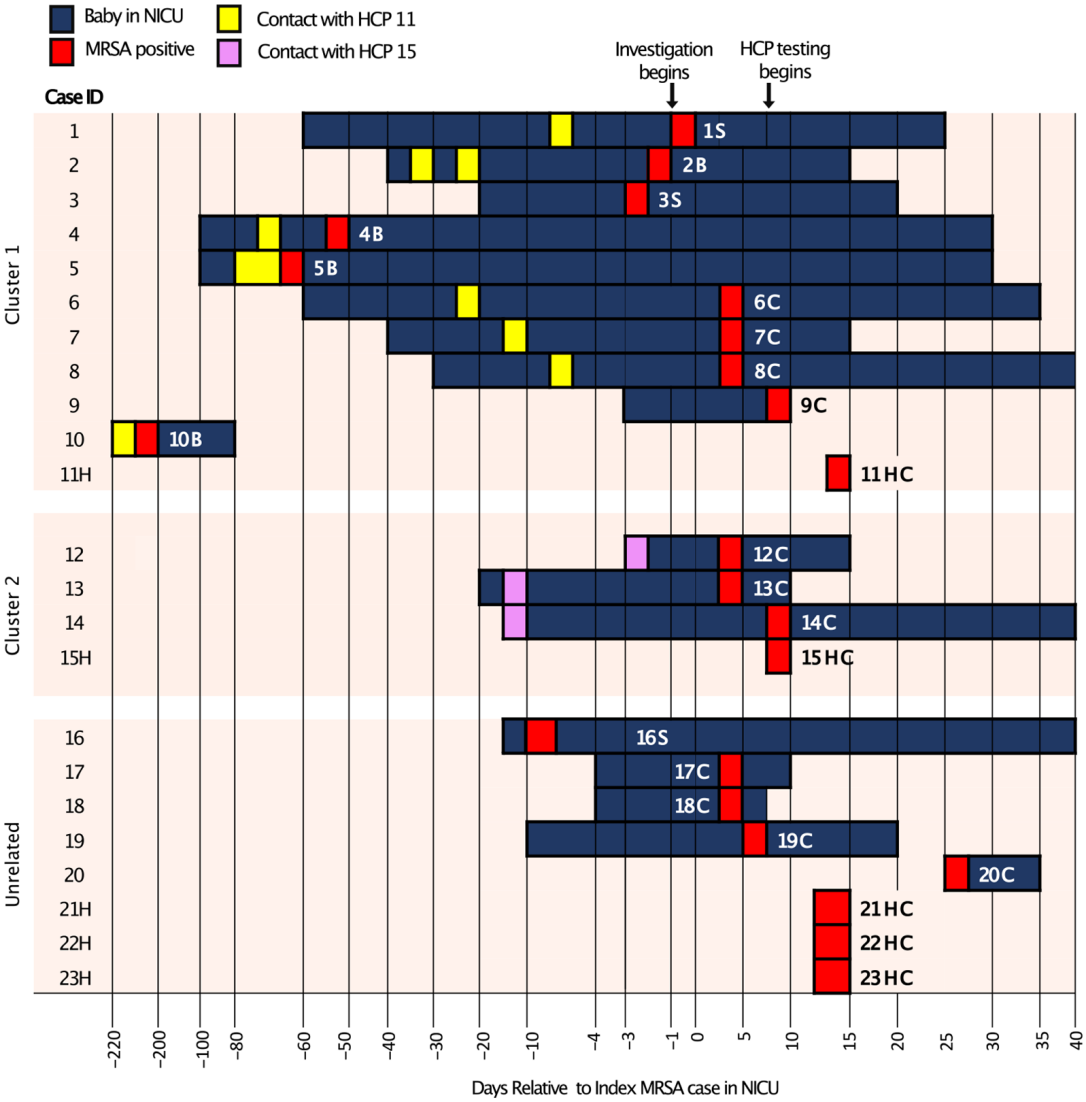
Epidemiologic Tracing of MRSA outbreak in the NICU. Index case and 22 other cases associated with an MRSA outbreak. Healthcare personnel (HCP) are indicated by an H. Epidemiologic link to each of 2 HCP is indicated by yellow (11H) and purple (15H), respectively.

Confirmation of a highly related cluster of MRSA isolates through WGS prompted further epidemiologic investigation that revealed that 8 of the 11 infants associated outbreak clade 1 had been cared for by HCP 11 (Fig. 2). HCP 11 also cared for patient 10B, who was identified 7 months prior to clade 1, raising the possibility that HCP 11 could be the source of the current outbreak. Almost all patients associated with the outbreak strains were born prematurely and exhibited low birthweight (Supplementary Table 1 online). None of the cases associated with outbreak clade 2 group exhibited active infection.

In silico MLST typing revealed that outbreak clade 1 was sequence type (ST)-8, and smaller outbreak clade 2 was ST-5 (Fig. 1B). Staphylococcal cassette chromosome (SCC) *mec* typing revealed further similarities among outbreak clades, with clade 1 samples identified to harbor SCC*mec* IVa, and clade 2 cases identified to harbor SCC*mec* V (Fig. 1B). Notably, MRSA isolate belonging to case ID 19C shared identical AST, MLST, and SCC*mec* typing as clade 1 isolates but was ~270 SNPs away from clade 1, and therefore was distinct from the predominant outbreak clade.

**Fig. 3.**
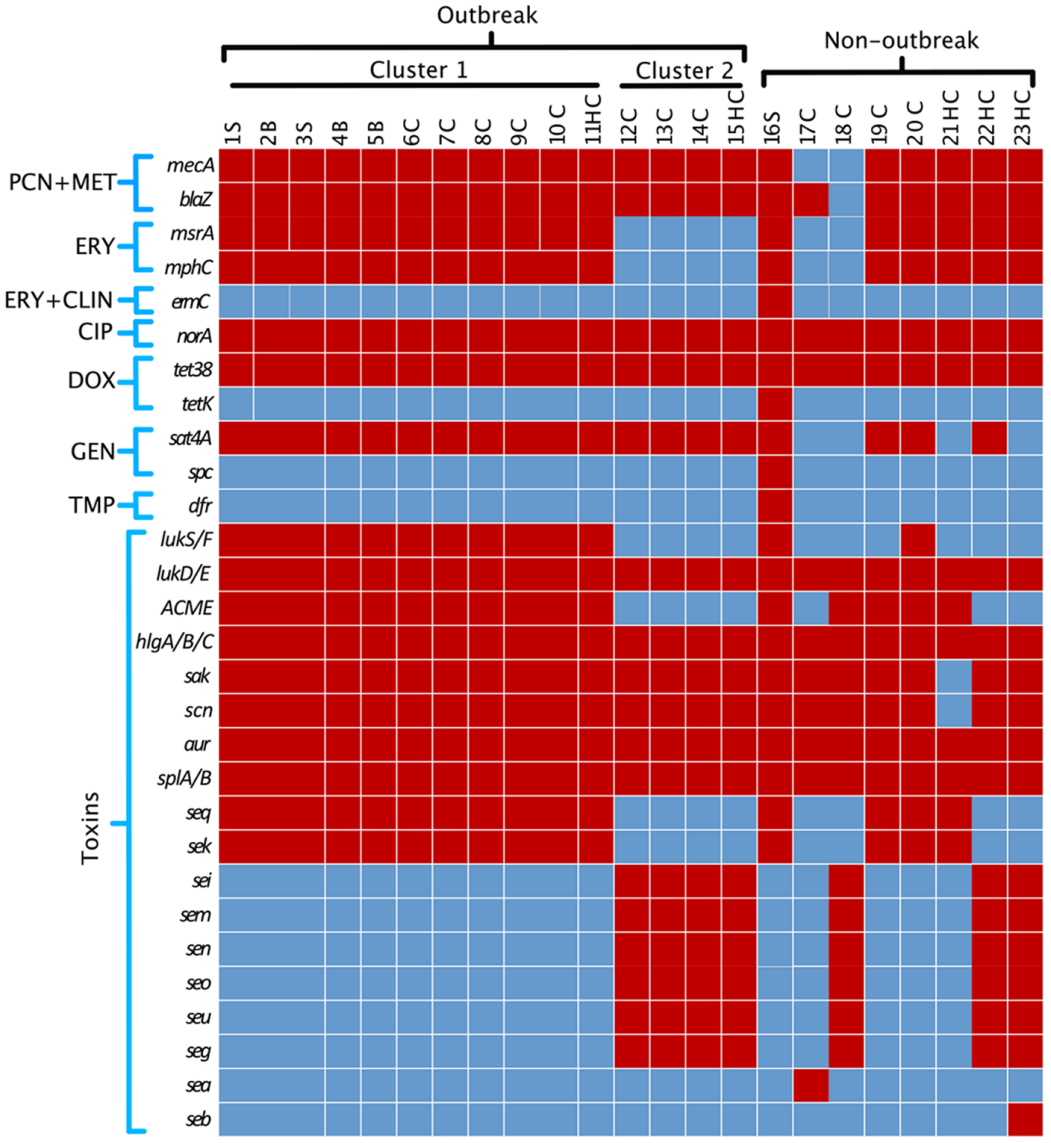
Antimicrobial resistance and toxin genes identified by WGS of MRSA isolates. Genes associated with antibiotic resistance and toxins are shown for all cases sequenced. Gene presence is depicted in red and gene absence is depicted in blue. Note. PCN, denotes penicillin; MET, methicillin; ERY, erythromycin; CLIN, clindamycin; CIP, ciprofloxacin; DOX, doxycycline; GEN, gentamycin; and TMP trimethoprim. Genes associated with toxins are shown.

Next, we carried out antimicrobial resistance, virulence, and toxin gene assessments. Cases associated with both outbreak clade 1 and clade 2 exhibited similar antimicrobial-resistance gene profiles (Fig. 3) and concordant phenotypic antimicrobial-resistance profile (Fig. 1B). Interestingly, across nonoutbreak-associated isolates, isolates 17C and 18C, lacked the *mec*A gene, associated with resistance to β-lactam antibiotics like methicillin and penicillin, despite being phenotypically resistant. Furthermore, 18C also lacked the *blaZ* gene, associated with resistance to penicillin, despite being phenotypically resistant. Harboring of aminoglycoside resistance genes, *sat4A* or *spc*, was not associated with phenotypic antimicrobial resistance.

Presence of genes encoding virulence factors or toxins revealed similarities across clade 1 and clade 2 isolates. Interestingly, clade 1 isolates harbored *lukS/F*, which encodes the cytotoxin Panton-Valentine leukocidin (PVL), and the arginine catabolic mobile element (ACME), which are associated with severe invasive infection^20^ or the promotion of persistence and skin colonization,^21^ respectively. Clade 1 also harbored superantigen enterotoxin genes *seq* and *sek*.^22^ In further contrast to clade 1, clade 2 isolates contained the enterotoxin gene clade *sei, sem, sen, seo, seg*, and *seu* encoded by superantigen enterotoxin gene clade *egc*.^23,24^ All isolates contained the gamma-hemolysin genes *hlgA/B/C* and the leukocidin genes *lukD/E*.

## Discussion

Real-time deployment of WGS provided high-resolution assessment of an outbreak involving dynamic exchange of genetically distinct MRSA strains between HCPs and patients in a NICU and allowed for strategic implementation of infection control measures. Phylogenetic analysis uncovered 2 outbreak clades in the NICU, both involving HCP who had contact with most, if not all, clade-associated isolates. Clade 1 was associated with invasive and severe infections, while clade 2 was associated with asymptomatic carriage.

Surprisingly, outbreak clade 1 included patient 10B, who was admitted to the NICU 7 months prior to the outbreak and was cared for by HCP 11, were also colonized by clade 1. This raises the possibility that HCP 11 may have played a role in the dissemination of the outbreak clade 1. Not all clade 1 isolates had an epidemiologic link to HCP 11, however, which raises the possibility of unrecognized inter-HCP transmission of the clade 1 strain or, alternatively, the presence of an environmental reservoir.

Assessment of *S. aureus* virulence factors in the strains from each clade suggested potential molecular determinants driving outbreak emergence. Clade 1 isolates associated with the pandemic clonal lineage USA300, classically regarded as a community-associated (CA-) MRSA strain. Clade 2 isolates most closely associated with strain NS315, a hospital-associated (HA-) MRSA strain. Both outbreak isolates harbored the *lukS/F* gene that encodes for the cytotoxin Panton-Valentine leukocidin (PVL), a protein first associated with epidemic CA-MRSA strains that are more likely to cause sepsis, necrotizing pneumonia, and necrotizing fasciitis.^20^ However, unlike clade 2, the clade 1 strain harbored the genomic island referred to as ACME. First described in CA-MRSA strain USA300 in 2006, ACME likely originated from *S. epidermidis*.^25^ This element is composed of 33 putative genes and 2 operons, *arc* and *opp*, which encode genes thought to confer increased survival in the acidic skin environment.^26^ Therefore, ACME-containing *S. aureus* are poised to execute epidemic, severe infections, possibly explaining why clade 1 isolate was able to circulate in the NICU for >9 months, causing multiple episodes of severe infection along the way. Rapid identification of potentially high-virulence strains highlights an opportunity for preventative surveillance of this high-risk population.

Notably, isolates 16P and 20C also harbored the *lukS/F* gene and the ACME element. These isolates were not associated with a clade and likely reflect singular acquisitions of highly virulent strains. Clade 1 and clade 2 isolates, on the other hand, had HCP to provide an epidemiologic link that could suggest repeated exposures to the highly virulent isolates that led to the pathologic infection and/or colonization of numerous neonates. This highlights the possibility that silent transmission of antimicrobial resistant organisms is taking place throughout the healthcare system, leaving our most vulnerable patients at greatest risk.

The use of WGS enabled earlier and definitive confirmation that an outbreak was occurring and was seminal to interpreting HCP MRSA surveillance results. Control of both outbreaks was obtained through several measures, including temporary closure of the NICU to new admissions, contact isolation (gown and gloves), positive case cohorting, reinforcement of hand hygiene and PPE practices, extensive environmental disinfection, and patient, parent, and HCP decolonization. All MRSA colonized patients and HCP underwent MRSA decolonization with nasal mupirocin (patients) or nasal povidone iodine (HCP) and topical chlorhexidine if they were eligible. The presence of an outbreak was disclosed to all families in the NICU and families of MRSA positive patients were offered counseling in addition to decolonization with nasal povidone iodine and topical chlorhexidine.

Several studies have suggested a higher prevalence of MRSA colonization in HCP compared to the general population,^27,28^ thus complicating the interpretation of HCP MRSA surveillance results. WGS permitted identification of HCP colonized with the outbreak clades, and subsequent targeted follow-up and decolonization protocols prior to their return to work. The precision monitoring enabled by WGS changed the ongoing infection control practices employed by our NICU by establishing prospective MRSA surveillance efforts. Ongoing surveillance in the NICU has not identified further clade 1 or 2 associated MRSA isolates 12 months after the outbreak investigation, underscoring the importance and efficacy of decolonization, as has been noted in the case of parental reservoirs.^29^

Although the directionality of MRSA transmission cannot be confirmed, both outbreak clades identified were associated with HCP through genetic and epidemiologic linkage. Prior MRSA outbreak studies in the NICU have also identified the potential contribution of the HCP reservoir,^10,30^ but few, if any, have shown prolonged circulation in the hospital environment through silent transmission events that have led to significant patient morbidity, as this work demonstrates. Although economic modeling studies of prospective genomic MRSA surveillance suggests its cost-effectiveness,^31^ assessment of HCP colonization through prospective screening may not prove feasible or practical. However, prospective genomic surveillance of high-risk patient populations, such as neonates, may prove beneficial in the early identification of silent and disease-associated outbreaks, as shown in this work.

Prospective genomic surveillance employed in this study was performed at a nonprofit academic research institute affiliated with our institution, in a laboratory that works closely with the hospital and infection prevention group. As the initial outbreak investigation unfolded, WGS was performed in small batches as positive isolated were identified. This resulted in a cost of ~$500 per sample, which includes cost for WGS from DNA extraction to data and laboratory staff time. Prospective genomic surveillance, however, can allow for larger batching of isolates and use of higher throughput sequencing platforms, thus further reducing costs. Given the wider availability of WGS strategies, and the higher cost-effectiveness of large batch sample processing, we believe that this model can be easily replicated by other hospital systems.

This study had several limitations. Although this work highlights potential that silent transmission of MRSA may occur for months before detection of an outbreak, we were unable to sequence all MRSA isolates detected in the NICU during the 7-month gap between sample the index case and the outbreak, nor isolates prior to the index case. This limited our ability to definitively pinpoint the introduction and complex transmission dynamics of outbreak clade 1. Further, the source of introduction of clade 1 and clade 2 remained elusive, which necessitated consideration of other potential reservoirs in addition to the affected HCPs, such as other HCPs, parents, and the environment. However, environmental samples were unavailable. Nevertheless, the high degree of genetic similarity of clade 1 isolates, evidenced by low SNP difference, suggests that clade 1 isolates were part of a singular transmission event and thus highlights the dangers of silent transmission of highly virulent isolates can pose.

In summary, our results suggest that transmission between HCP and patients contributed to a NICU outbreak involving a uniquely virulent MRSA strain. WGS enabled data-driven implementation of infection prevention strategies that prevented transmission of the outbreak strains and identified a potential source. Prospective genomic epidemiology of hospital-acquired infections may help identify occult transmission events that may precede outbreaks of MRSA and other pathogens. Our findings corroborate prior work demonstrating the utility of WGS in outbreak management.^18,32,33^ Given the increasing availability and affordability of WGS, genomic epidemiology of CDC priority pathogens from staff and patients may be a useful measure for identifying early colonization and silent transmission events of virulent strains prior to the emergence of an outbreak.

## Supplementary Material

Supp1

Supp2

**Supplementary material.** To view supplementary material for this article, please visit https://doi.org/10.1017/ice.2022.48

## References

[1] Lake JG, Weiner LM, Milstone AM, Saiman L, Magill SS, See I. Pathogen distribution and antimicrobial resistance among pediatric healthcare-associated infections reported to the National Healthcare Safety Network, 2011–2014. Infect Control Hosp Epidemiol 2018;39:1–11. 29249216 10.1017/ice.2017.236 PMC6643994

[2] Shah J, Jefferies A, Yoon E, Lee S, Shah P, on behalf of the Canadian Neonatal Network. Risk factors and outcomes of late-onset bacterial sepsis in preterm neonates born at <32 weeks’ gestation. Am J Perinatol 2014;32:675–682. 25486288 10.1055/s-0034-1393936

[3] Song X, Perencevich E, Campos J, Short BL, Singh N. Clinical and economic impact of methicillin-resistant *Staphylococcus aureus* colonization or infection on neonates in intensive care units. Infect Control Hosp Epidemiol 2010;31:177–182. 20001732 10.1086/649797

[4] Zervou FN, Zacharioudakis IM, Ziakas PD, Mylonakis E. MRSA colonization and risk of infection in the neonatal and pediatric ICU: a meta-analysis. Pediatrics 2014;133:e1015–e1023. 24616358 10.1542/peds.2013-3413

[5] Wagenvoort JHT, Sluijsmans W, Penders RJR. Better environmental survival of outbreak vs. sporadic MRSA isolates. J Hosp Infect 2000;45:231–234. 10896803 10.1053/jhin.2000.0757

[6] Jimenez-Truque N, Tedeschi S, Saye EJ, Relationship between maternal and neonatal *Staphylococcus aureus* colonization. Pediatrics 2012;129: e1252–e1259. 22473373 10.1542/peds.2011-2308 PMC3340589

[7] Hollis RJ, Barr JL, Doebbeling BN, Pfaller MA, Wenzel RP. Familial carriage of methicillin-resistant *Staphylococcus aureus* and subsequent infection in a premature neonate. Clin Infect Dis 1995;21:328–332. 8562740 10.1093/clinids/21.2.328

[8] Al-Tawfiq JA. Father-to-infant transmission of community-acquired methicillin-resistant *Staphylococcus aureus* in a neonatal intensive care unit. Infect Control Hosp Epidemiol 2006;27:636–637. 16755488 10.1086/505097

[9] Boyce JM, Opal SM, Potter-Bynoe G, Medeiros AA. Spread of methicillin-resistant *Staphylococcus aureus* in a hospital after exposure to a healthcare worker with chronic sinusitis. Clin Infect Dis 1993;17:496–504. 8218696 10.1093/clinids/17.3.496

[10] Saiman L, Cronquist A, Wu F, An outbreak of methicillin-resistant *Staphylococcus aureus* in a neonatal intensive care unit. Infect Control Hosp Epidemiol 2003;24:317–321. 12785403 10.1086/502217

[11] Bertin ML, Vinski J, Schmitt S, Outbreak of methicillin-resistant *Staphylococcus aureus* colonization and infection in a neonatal intensive care unit epidemiologically linked to a healthcare worker with chronic otitis. Infect Control Hosp Epidemiol 2006;27:581–585. 16755477 10.1086/504933

[12] Kamm J. SPID: SNP pipeline for infectious disease. https://github.com/czbiohub/Spid.jl. Accessed February 28, 2021.

[13] Stamatakis A. RAxML version 8: a tool for phylogenetic analysis and postanalysis of large phylogenies. Bioinformatics 2014;30:1312–1313. 24451623 10.1093/bioinformatics/btu033 PMC3998144

[14] Huerta-Cepas J, Serra F, Bork P. ETE 3: reconstruction, analysis, and visualization of phylogenomic data. Mol Biol Evol 2016;33:1635–1638. 26921390 10.1093/molbev/msw046 PMC4868116

[15] Gupta SK, Padmanabhan BR, Diene SM, ARG-ANNOT, a new bio-informatic tool to discover antibiotic resistance genes in bacterial genomes. Antimicrob Agents Chemother 2014;58:212–220. 24145532 10.1128/AAC.01310-13 PMC3910750

[16] Kaya H, Hasman H, Larsen J, SCCmecFinder, a web-based tool for typing of staphylococcal cassette chromosome mec in *Staphylococcus aureus* using whole-genome sequence data. mSphere 2018;3:e00612–17. 29468193 10.1128/mSphere.00612-17 PMC5812897

[17] Larsen MV, Cosentino S, Rasmussen S, Multilocus sequence typing of total-genome–sequenced bacteria. J Clin Microbiol 2012;50: 1355–1361. 22238442 10.1128/JCM.06094-11 PMC3318499

[18] Joensen KG, Scheutz F, Lund O, Real-time whole-genome sequencing for routine typing, surveillance, and outbreak detection of verotoxigenic *Escherichia coli*. J Clin Microbiol 2014;52:1501–1510. 24574290 10.1128/JCM.03617-13 PMC3993690

[19] Uhlemann A-C, Dordel J, Knox JR, Molecular tracing of the emergence, diversification, and transmission of *S. aureus* sequence type 8 in a New York community. Proc Natl Acad Sci U S A 2014;111: 6738–6743. 24753569 10.1073/pnas.1401006111 PMC4020051

[20] Gillet Y, Issartel B, Vanhems P, Association between *Staphylococcus aureus* strains carrying gene for Panton-Valentine leukocidin and highly lethal necrotising pneumonia in young immunocompetent patients. Lancet 2002;359:753–759. 11888586 10.1016/S0140-6736(02)07877-7

[21] Planet PJ, LaRussa SJ, Dana A, Emergence of the epidemic methicillin-resistant *Staphylococcus aureus* strain USA300 coincides with horizontal transfer of the arginine catabolic mobile element and speG-mediated adaptations for survival on skin. mBio 2013;4:e00889–13. 24345744 10.1128/mBio.00889-13 PMC3870260

[22] Yarwood JM, McCormick JK, Paustian ML, Orwin PM, Kapur V, Schlievert PM. Characterization and expression analysis of *Staphylococcus aureus* pathogenicity island 3: implications for the evolution of staphylococcal pathogenicity islands. J Biol Chem 2002;277:13138–13147. 11821418 10.1074/jbc.M111661200

[23] Kuroda M, Ohta T, Uchiyama I, Whole-genome sequencing of meticillin-resistant *Staphylococcus aureus*. Lancet 2001;357:1225–1240. 11418146 10.1016/s0140-6736(00)04403-2

[24] Letertre C, Perelle S, Dilasser F, Fach P. Identification of a new putative enterotoxin SEU encoded by the egc cluster of *Staphylococcus aureus*. J Appl Microbiol 2003;95:38–43. 12807452 10.1046/j.1365-2672.2003.01957.x

[25] Diep BA, Gill SR, Chang RF, Complete genome sequence of USA300, an epidemic clone of community-acquired meticillin-resistant *Staphylococcus aureus*. Lancet 2006;367:731–739. 16517273 10.1016/S0140-6736(06)68231-7

[26] Thurlow LR, Joshi GS, Clark JR, Functional modularity of the arginine catabolic mobile element contributes to the success of USA300 methicillin-resistant *Staphylococcus aureus*. Cell Host Microbe 2013;13:100–107. 23332159 10.1016/j.chom.2012.11.012 PMC3553549

[27] da Silva LSC, Andrade YMFS, Oliveira AC, Prevalence of methicillin-resistant *Staphylococcus aureus* colonization among healthcare workers at a tertiary-care hospital in northeastern Brazil. Infect Prev Pract 2020;2:100084. 34368723 10.1016/j.infpip.2020.100084 PMC8336055

[28] Elie-Turenne M-C, Fernandes H, Mediavilla JR, Prevalence and characteristics of *Staphylococcus aureus* colonization among healthcare professionals in an urban teaching hospital. Infect Control Hosp Epidemiol 2010;31:574–580. 20426580 10.1086/652525

[29] Milstone AM, Voskertchian A, Koontz DW, Effect of treating parents colonized with *Staphylococcus aureus* on transmission to neonates in the intensive care unit: a randomized clinical trial. JAMA 2020;323:319. 31886828 10.1001/jama.2019.20785 PMC6990934

[30] Bertin ML, Vinski J, Schmitt S, Outbreak of methicillin-resistant *Staphylococcus aureus* colonization and infection in a neonatal intensive care unit epidemiologically linked to a healthcare worker with chronic otitis. Infect Control Hosp Epidemiol 2006;27:581–585. 16755477 10.1086/504933

[31] Dymond A, Davies H, Mealing S, Genomic surveillance of methicillin-resistant *Staphylococcus aureus*: a mathematical early modeling study of cost-effectiveness. Clin Infect Dis 2020;70:1613–1619. 31219153 10.1093/cid/ciz480 PMC7145999

[32] Snitkin ES, Zelazny AM, Thomas PJ, Tracking a hospital outbreak of carbapenem-resistant *Klebsiella pneumoniae* with whole-genome sequencing. Sci Transl Med 2012;4:148ra116. 10.1126/scitranslmed.3004129 PMC3521604 22914622

[33] Crawford E, Kamm J, Miller S, Investigating transfusion-related sepsis using culture-independent metagenomic sequencing. Clin Infect Dis 2020;71:1179–1185. 31563940 10.1093/cid/ciz960 PMC7442849

