# Supplementary material for: Prolonged silent carriage, genomic virulence potential and transmission between staff and patients characterize a neonatal intensive care unit (NICU) outbreak of methicillin-resistant Staphylococcus aureus (MRSA)
Source: Infect Control Hosp Epidemiol. Author manuscript; Available in PMC 2026 Feb 17. (PMC12911661; doi:10.1017/ice.2022.48)
Supplement: Supp1 [file NIHMS2139428-supplement-Supp1.pdf]

ID	Ciprofloxacin	Clindamycin	Penicillin	Methicillin	Erythromycin	Tetracycline	Gentamycin	TMP–SMX	Vancomycin
1S	R		R	R	R				
2B	R		R	R	R				
3S	R		R	R	R				
4B	R		R	R	R				
5B	R		R	R	R				
6C	R		R	R	R				
7C	R		R	R	R				
8C	R		R	R	R				
9C	R		R	R	R				
10B	R		R	R	R				
11HC	R		R	R	R				
12C			R	R					
13C			R	R					
14C			R	R					
15HC			R	R					
16S	R	R	R	R	R	R			
17C			R	R	I				
18C	R	R	R	R	R		I		
19C	R		R	R	R				
20C			R	R	R				
21HC			R	R					
22HC	R		R	R	R				
23HC	R		R	R					
