# Supplementary material for: Prolonged silent carriage, genomic virulence potential and transmission between staff and patients characterize a neonatal intensive care unit (NICU) outbreak of methicillin-resistant Staphylococcus aureus (MRSA)
Source: Infect Control Hosp Epidemiol. Author manuscript; Available in PMC 2026 Feb 17. (PMC12911661; doi:10.1017/ice.2022.48)
Supplement: Supp2 [file NIHMS2139428-supplement-Supp2.pdf]

| First Positive Culture* | ID | Sex | GA at birth | Birthweight (g) | Reason for ICU Admission | MRSA Manifestation | Site |
|---|---|---|---|---|---|---|---|
| -220 | 10B | Female | 29w6d | 1515 | Prematurity, RDS | Bacteremia, Meningitis | Blood |
| -65 | 5B | Male | 27w1d | 935 | Prematurity, RDS, PDA, PFO | Bacteremia | Blood |
| -55 | 4B | Male | 27w1d | 1395 | Prematurity, RDS, PDA | Bacteremia | Blood |
| -8 | 16S | Male | 31w6d | 1572 | Prematurity, RDS | SSTI | Wound |
| -3 | 3S | Male | 33w1d | 2175 | Prematurity, RDS, Congenital Syphilis | SSTI | Wound |
| -1 | 2B | Female | 32w5d | 1840 | Prematurity, Scimitar Syndrome, Aortic coarctation, VSD, pTHN | Bacteremia | Blood |
| 0 | 1S | Female | 25w0d | 800 | Prematurity, RDS | SSTI | Wound |
| 3 | 6C | Female | 32w1d | 1420 | Prematurity, Omphalocele | Colonization | Nares⁺ |
| 3 | 7C | Male | 33w0d | 2760 | Prematurity, Myelomeningocele, | Colonization | Nares⁺ |
| 3 | 8C | Male | 26w0d | 830 | Prematurity, Perforated Viscus | Colonization | Nares⁺ |
| 3 | 12C | Male | 26w0d | 1675 | Prematurity, RDS | Colonization | Nares⁺ |
| 3 | 13C | Male | 28w0d | 1330 | Prematurity, RDS | Colonization | Nares⁺ |
| 3 | 17C | Male | 40w0d | 3485 | PPHN | Colonization | Nares⁺ |
| 3 | 18C | Male | 30w1d | 1700 | Prematurity, RDS, HIE | Colonization | Nares⁺ |
| 5 | 19C | Female | 39w1d | 3845 | dTGA, VSD | Colonization | Nares⁺ |
| 8 | 9C | Male | 28w0d | 1300 | Prematurity, RDS | Colonization | Nares⁺ |
| 8 | 14C | Male | 28w6d | 1030 | Prematurity, RDS | Colonization | Nares⁺ |
| 10 | 15HC | | | | | Colonization | Nares⁺ |
| 15 | 11HC | | | | | Colonization | Nares⁺ |
| 15 | 21HC | | | | | Colonization | Nares⁺ |
| 15 | 22HC | | | | | Colonization | Nares⁺ |
| 16 | 23HC | | | | | Colonization | Nares⁺ |
| 26 | 20C | Male | 39w2d | 3840 | Tetralogy of Fallot | Colonization | Nares⁺ |

* days relative to index MRSA case in the NICU
⁺ Nares and sites defined in Methods section
